# Genetically Determined Inflammatory Biomarkers and the Risk of Heart Failure: A Mendelian Randomization Study

**DOI:** 10.3389/fcvm.2021.734400

**Published:** 2021-11-22

**Authors:** Xintao Li, Shi Peng, Bo Guan, Songwen Chen, Genqing Zhou, Yong Wei, Chao Gong, Juan Xu, Xiaofeng Lu, Xiaoyu Zhang, Shaowen Liu

**Affiliations:** ^1^Department of Cardiology, Shanghai General Hospital, Shanghai Jiao Tong University School of Medicine, Shanghai, China; ^2^Geriatric Cardiology Department of the Second Medical Center and National Clinical Research Center for Geriatric Diseases, Chinese PLA General Hospital, Beijing, China; ^3^Beijing Key Laboratory of Clinical Epidemiology, School of Public Health, Capital Medical University, Beijing, China; ^4^Department of Anesthesiology, Sanbo Brain Hospital, Capital Medical University, Beijing, China

**Keywords:** heart failure, Mendelian randomization, C-reactive protein, fibrinogen, interleukin

## Abstract

**Background:** Positive associations between inflammatory biomarkers and the risk of heart failure (HF) have been reported in conventional observational studies. However, the causal effects of inflammatory biomarkers on HF have not been fully elucidated. We conducted a Mendelian randomization (MR) study to examine the possible etiological roles of inflammatory biomarkers in HF.

**Methods:** Summary statistical data for the associations between single nucleotide polymorphisms (SNPs) and C-reactive protein (CRP), fibrinogen, and components of the interleukin-1 (IL-1)-interleukin-6 (IL-6) inflammatory signaling pathway, namely, interleukin-1β (IL-1β), IL-1 receptor antagonist (IL-1ra), IL-6, and soluble IL-6 receptor (sIL-6r), were obtained from genome-wide association studies (GWASs) for individuals of European descent. The GWAS dataset of 977,323 participants of European ancestry, which included 47,309 HF cases and 930,014 controls, was collected to identify genetic variants underlying HF. A two-sample Mendelian randomization framework was implemented to examine the causality of the association between these inflammatory biomarkers and HF.

**Results:** Our MR analyses found that genetically determined CRP and fibrinogen were not causally associated with HF risk (odds ratio [OR] = 0.93, 95% confidence interval [CI] = 0.84–1.02, *p* = 0.15; OR = 0.94, 95% CI = 0.55–1.58, *p* = 0.80, respectively). These findings remained consistent using different Mendelian randomization methods and in sensitivity analyses. For the IL-1-IL-6 pathway, causal estimates for IL-6 (OR = 0.86, 95% CI 0.81–0.91, *p* < 0.001), but not for IL-1β, IL-1ra, or sIL-6r, were significant. However, the association between genetically determined IL-6 and HF risk became non-significant after excluding SNPs with potential pleiotropy (OR = 0.89, 95% CI = 0.77–1.03, *p* = 0.12).

**Conclusion:** Our study did not identify convincing evidence to support that CRP and fibrinogen, together with their upstream IL-1-IL-6 signaling pathway, were causally associated with HF risk.

## Introduction

Heart failure, a debilitating condition in which the heart fails to respond to increased cardiac output to meet peripheral demands, is a worldwide health burden (1). Currently, ~1–2% of adult populations in developed countries are victims of heart failure (2). Given the prolonged life expectancy of the general population and the vulnerability of the elderly to cardiac dysfunction (3), the prevalence of HF is predicted to have a two-fold increase by 2060 (4). Therefore, exploring underlying pathophysiological mechanisms to identify therapeutic targets for improving HF prognosis is a clinically unmet need.

Inflammation has been implicated in the pathogenesis of heart failure (HF). Previously, observational studies have reported that C-reactive protein (CRP), a representative biomarker of systemic inflammation, can predict the development and prognosis of HF (5–7). Another inflammatory marker, fibrinogen, is a vital determinant of blood viscosity and platelet aggregation, and has also been suggested to be associated with HF risk (8, 9). However, residual confounding and reverse causality may remain alternative explanations for the strong association between CRP and fibrinogen with heart failure because of the inherent limitation of conventional observational studies (10).

“Upstream” proinflammatory cytokines, such as interleukin-1 (IL-1) and interleukin-6 (IL-6), are major initiators for the production of “downstream” biomarkers, such as CRP and fibrinogen, from the liver (11, 12). Established associations between the classic IL-1-IL6-CRP signaling and adverse cardiac remodeling have led to investigations targeting this pathway to reduce inflammation and alleviate cardiac dysfunction (13). However, contradictory results from HF therapy based on anti-inflammation suggested otherwise (14, 15). High serum levels of IL-1 receptor antagonist (IL-1ra) and soluble IL-6 receptor (sIL-6r) have been reported to mimic the effects of endogenous inhibition of IL-1 and IL-6 signaling, respectively (16). However, investigation of these proximal inflammatory mediators is difficult given that their concentrations are subjected to constant fluctuations in the bloodstream.

Mendelian randomization is a form of analysis that uses genetic variants as instrumental variables (IVs) to generate more reliable causal estimates of long-exposure effects of risk factors on disease outcomes (17). This approach takes advantage of the naturally occurring random allocation of alleles at conception (18). Therefore, the level of a specific exposure will usually be independent of other exposures and unaffected by disease status (19). Thus, Mendelian randomization (MR) is capable of overcoming the limitations of residual confounding and reverse causation in observational studies. Here, we perform a two-sample MR analysis to test the hypothesis that genetically determined CRP and fibrinogen and their upstream inflammatory biomarkers, IL-1 and IL-6, are associated with HF risk in a European population.

## Methods

### Study Design and Data Source

**Figure 1:**
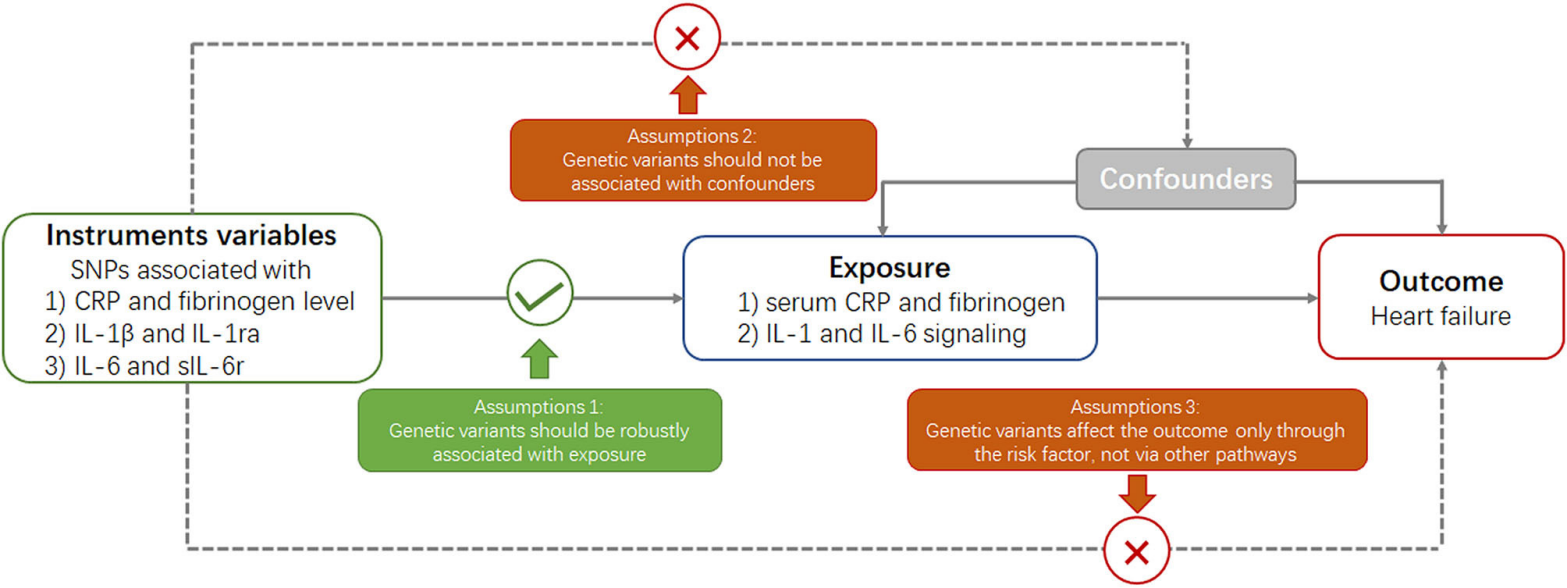
Key assumptions underlying Mendelian randomization study design. CRP, C-reactive protein; SNP, single nucleotide polymorphism.

Mendelian randomization is built upon three main assumptions (Figure 1) (20). First, genetic variants selected as instrumental variables should be robustly associated with the risk factor. Second, no association should exist between genetic variants and confounders. Third, genetic variants should affect the risk of outcome through the risk factor and not *via* other pathways.

**Figure 2:**
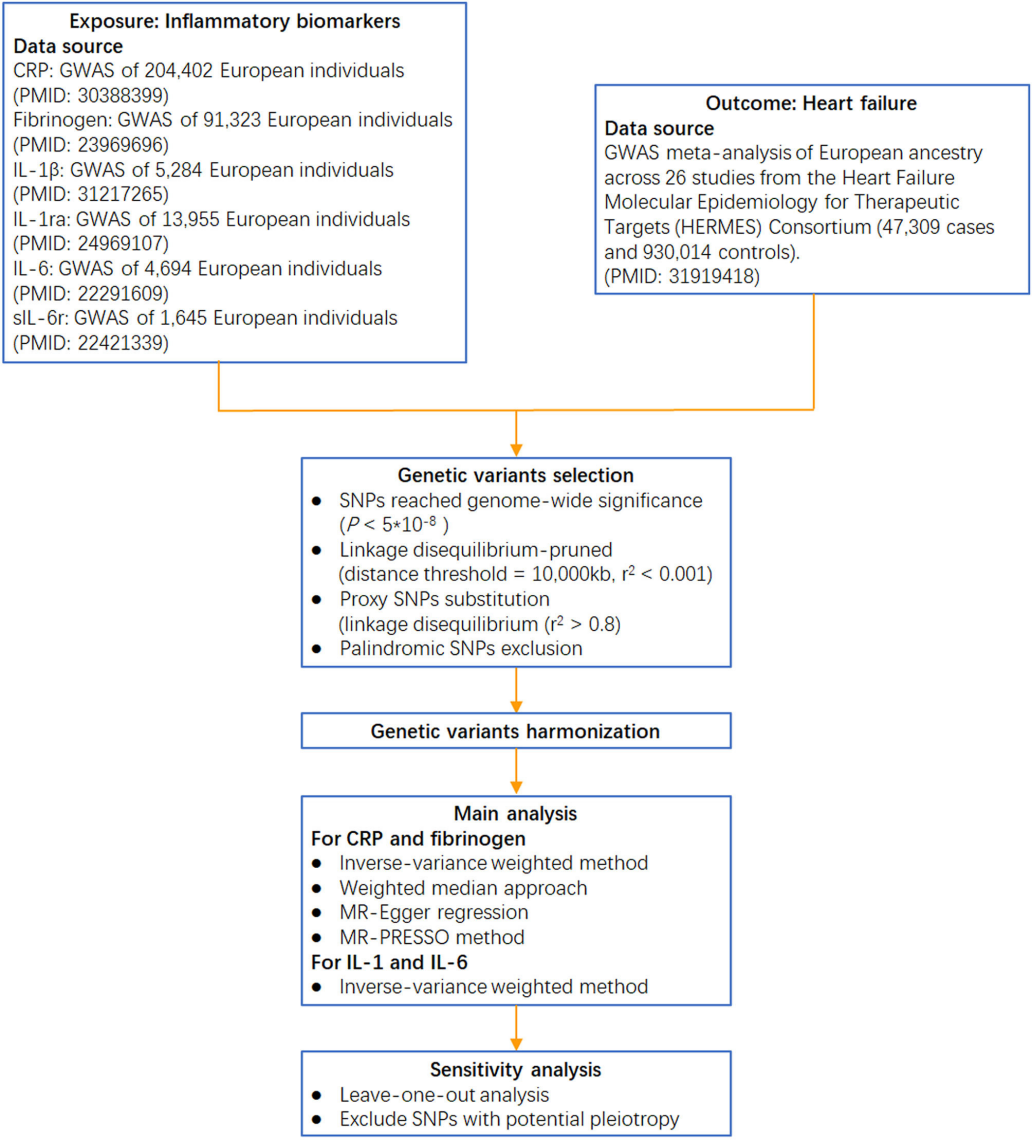
Flow chart of this Mendelian randomization study. IL-1β: interleukin-1β; IL-1ra: interleukin-1 receptor antagonist; sIL-6r: soluble IL-6 receptor; GWAS: genome-wide association study.

Circulating CRP-associated variants were collected from the largest genome-wide association study (GWAS) aimed at identifying variants in relation to CRP concentration (involving 204,402 individuals from 88 population-based cohort studies) (21). Genetic variants for fibrinogen were identified from a GWAS meta-analysis, which enrolled >90,000 individuals from 28 studies (22). Genetic instruments for IL-1β were obtained from a GWAS of the Northern Finland Birth Cohort (23), and those for IL-1ra were obtained from a GWAS meta-analysis of 11 cohorts (24). Genetic variants for IL-6 were collected from a GWAS of the SardiNIA project (25), and those for sIL-6r were collected from a collaborative meta-analysis of human genetic and biomarker data (26) (the analytical procedure is presented in Figure 2).

Following the identification of the genetic variants, summary statistical data on the association of genetic variants with heart failure were extracted from the published GWAS performed by the Heart Failure Molecular Epidemiology for Therapeutic Targets (HERMES) Consortium on 47,309 cases and 930,014 controls (27). HF cases from 26 cohorts of the HERMES Consortium were identified based on the clinical diagnosis of HF of any etiology with no specific inclusion criteria for left ventricular (LV) ejection fraction (Supplementary Table S1). Details of participant selection can be found elsewhere (27).

The datasets used in our study included individuals of European ancestry to reduce selection bias and to improve the robustness of the analysis. All the data used in this study were derived from GWAS for which ethical approval and patient consent were previously obtained. The study protocols were in accordance with the guidelines of the Helsinki Declaration and approved by the ethics committee of all participating sites.

### Genetic Variant Selection

Single-nucleotide polymorphisms that reached genome-wide significance (*p* < 5 ^*^ 10^−8^) were selected as instrumental variables. These SNPs were further linkage disequilibrium (LD)-pruned (distance threshold = 10,000 kb, *r*^2^ < 0.001) to ensure independence among the genetic variants (28). If the selected SNPs were not collected in the GWAS of HF, proxy SNPs in the linkage disequilibrium (*r*^2^ > 0.8) were chosen for substitution. Subsequently, palindromic SNPs were removed to ensure that the effects of the SNPs on the exposure corresponded to the same allele as their effects on HF. Accordingly, for CRP and fibrinogen, 41 and 19 SNPs, respectively, were included in the primary analysis of the association with HF. One SNP for IL-1β (rs6917603), two SNPs for IL-1ra (rs4251961, rs6759676), two SNPs for IL-6 (rs4129267, rs643434), and one SNP for sIL-6r (rs2228145) were also enrolled. The phenotypic variance explained by the selected SNPs was ~6.5% for CRP variation, 3.7% for fibrinogen variation, 1% for IL-1β, 2% for IL-1ra, 0.6% for IL-6, and 4% for sIL-6r.

### Statistical Analysis

We first harmonized the summary exposure and outcome data based on a previously described method (Supplementary Table S2) (29). Then, for CRP- and fibrinogen-associated instruments, four different methods of two-sample MR, namely, inverse-variance weighted, weighted median, MR-Egger, and MR-PRESSO, were implemented to calculate estimates and address possible heterogeneity and horizontal pleiotropy across the causal estimates (30). We used a predefined decision tree to select the best statistical estimation from the four methods as previously described (Supplementary Figure S1) (18). For biomarkers of IL-1β, IL-1ra, IL-6, and sIL-6r, the Wald ratio method was used to calculate estimates by dividing the beta coefficient for the SNP-outcome association with the beta coefficient for the SNP-biomarker effect. The inverse-variance weighted method with fixed effects was used to combine the Wald estimates for two SNPs.

To test the reliability of causal effect estimates, several sensitivity analyses were performed. First, a leave-one-out analysis was further conducted by removing a single variant from the analysis each time to determine whether the influence of a single SNP disproportionately affected the association. An additional sensitivity analysis was performed to address horizontal pleiotropic bias by excluding any SNPs significantly associated with potential confounders, such as body mass index (31), type 2 diabetes (32), systolic blood pressure (33), diastolic blood pressure (33), high-density lipoprotein cholesterol (34), low-density lipoprotein cholesterol (34), triglyceride (34), and smoking (35).

All results are presented as odds ratios (ORs) and corresponding 95% confidence intervals (CIs) of the outcomes with per predicted increase in CRP and fibrinogen concentrations (36). A two-sided *P* < 0.05 was defined as statistically significant. All the analyses were performed with the TwoSampleMR and MR-PRESSO packages with R version 4.0.2.

## Results

### Causal Effect of CRP and Fibrinogen on Heart Failure

**Figure 3:**
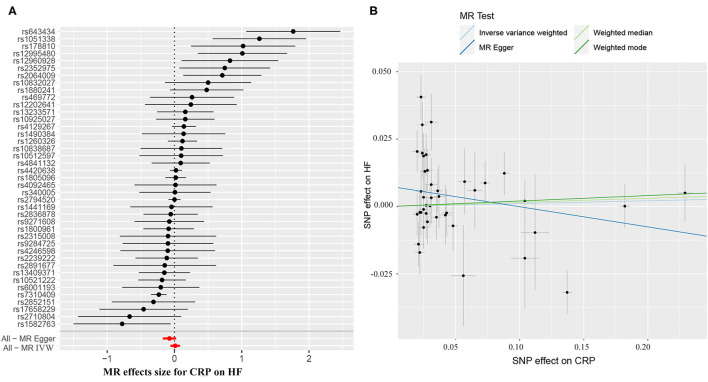
**(A)** Forest plot and **(B)** scatter plot of the potential effects of CRP-associated SNPs on heart failure (HF). IVW, inverse variance weighted. MR, Mendelian randomization.

**Figure 4:**
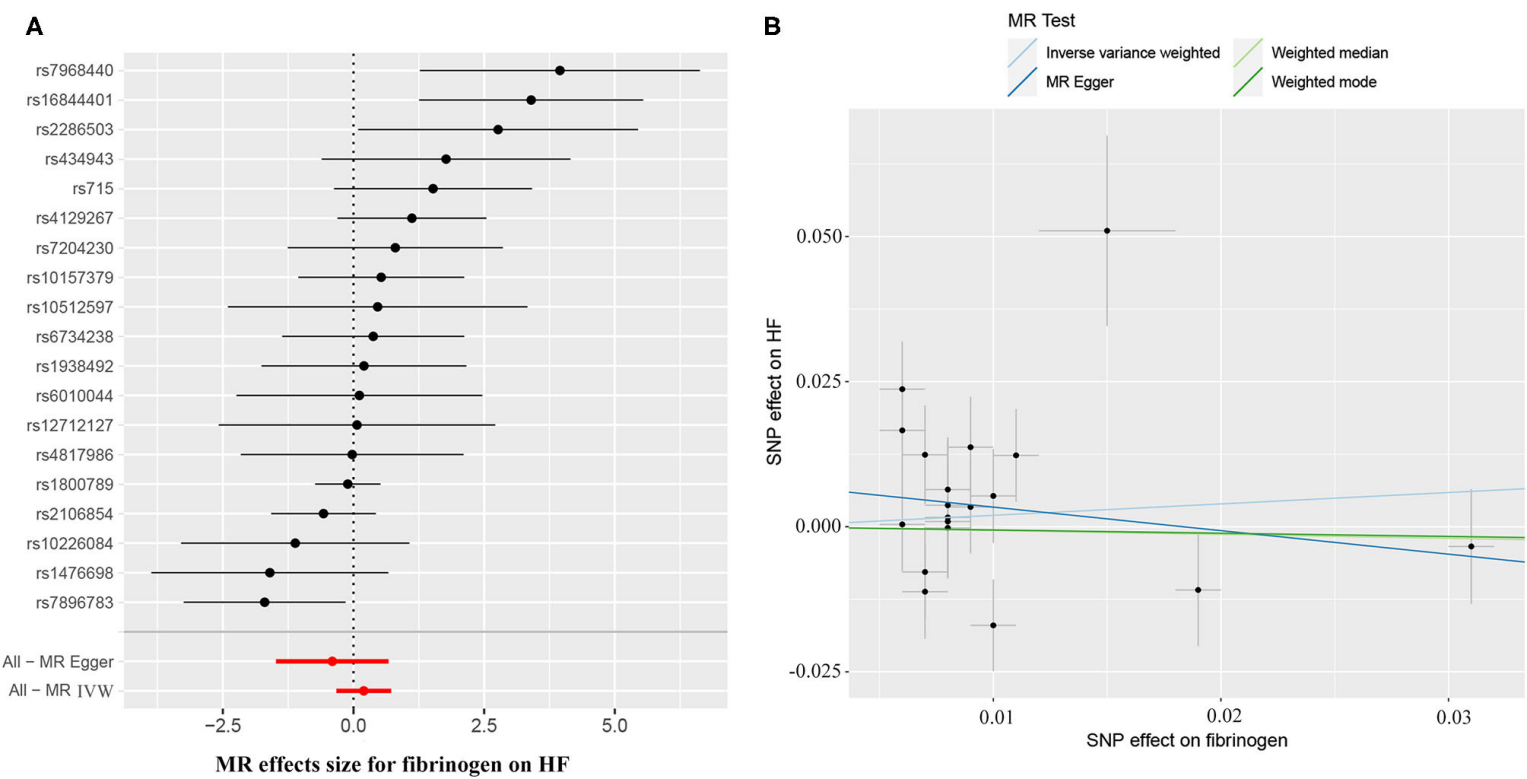
**(A)** Forest plot and **(B)** scatter plot of the potential effects of fibrinogen-associated SNPs on HF.

The causal effect estimates of genetically determined CRP and fibrinogen on the risk of HF are presented in Figures 3, 4, respectively. Significant heterogeneity was detected by the Cochran heterogeneity test among SNPs of CRP (*Q* = 108.8; *p* < 0.001) and fibrinogen (*Q* = 38.2; *p* = 0.004). The MR-Egger method showed significant directional pleiotropy for the association of CRP with HF [odds (intercept), 0.0076; *p* = 0.025] but not for fibrinogen [odds (intercept), 0.0074; *p* = 0.229]. Thus, based on a predefined decision tree (Supplementary Figure S1), no significant causal effects between CRP (MR Egger; OR = 0.93, 95% CI = 0.84–1.02, *p* = 0.15) and fibrinogen (weighted median; OR = 0.94, 95% CI = 0.55–1.58, *p* = 0.80) and HF risk were observed (Table 1). The lack of causal association persisted using all of the MR methods employed here. Leave-one-out sensitivity analyses did not reveal any significant change (Supplementary Figure S2). After excluding SNPs with potential pleiotropy, 13 SNPs for CRP and 11 SNPs for fibrinogen remained for analysis, and the results confirmed that neither genetically predicted CRP levels (weighted median; OR = 1.01, 95% CI = 0.93–1.09, *p* = 0.83) nor fibrinogen concentration (IVW, OR = 1.18, 95% CI = 0.77–1.81, *p* = 0.46) was associated with HF risk (Supplementary Table S3; Supplementary Figure S3).

**Table 1:** Mendelian randomization (MR) estimates of CRP and fibrinogen with heart failure.

**Phenotype and methods**	**IVs (SNPs)**	**OR (95% CI)**	***P*** **-value**	***Q*** **-statistics**	***P*** **h**
**CRP**					
IVW	41	1.01 (0.94–1.09)	0.77	108.8	<0.001
Weighted median	41	1.02 (0.95–1.08)	0.64		
**MR-Egger**	41	0.93 (0.84–1.02)	0.15		
MR-PRESSO	37	1.03 (0.98–1.10)	0.21		
**Fibrinogen**					
IVW	19	1.21 (0.72–2.06)	0.46	38.2	0.004
**Weighted median**	19	0.94 (0.55–1.58)	0.80		
MR-Egger	19	0.67 (0.23–1.96)	0.15		
MR-PRESSO	19	1.05 (0.62–1.77)	0.85		

*CRP, C-reactive protein; IV, instrumental variable; OR, odds ratio; CI, confidence interval; Ph, P for heterogeneity*.

### Causal Association Between the IL-1-IL-6 Pathway and Heart Failure

The results of the causal association between IL-1 and IL-6 signaling and HF are presented in Table 2. For IL-1 signaling, one SNP (rs6917603) was selected for IL-1β, and two SNPs (rs4251961 and rs6759676) were selected for IL-1ra. For IL-6 signaling, two SNPs were selected for IL-6 (rs4129267, rs643434), and one SNP was selected for sIL-6r (rs2228145). Primary MR analyses revealed an inverse causal effect of IL-6 on HF risk (OR = 0.86, 95% CI 0.81–0.91, *p* < 0.001), whereas no significant association between genetically determined IL-1β (OR = 1.04, 95% CI 0.86–1.25, *p* = 0.70), IL-1ra (OR = 0.95, 95% CI 0.83–1.10, *p* = 0.51), and sIL-6r (OR = 0.96, 95% CI 0.91–1.01, *p* = 0.14) with HF development was observed (Table 2). However, strong pleiotropic associations were noted between rs4251961 and rs643434 and cardiometabolic traits. After excluding the SNPs with pleiotropy, one SNP remained for IL-1ra and one for IL-6, and no significant causal effect of the IL-1-IL-6 pathway components on HF risk was observed (Table 2).

**Table 2:** Association between IL-1 and IL-6 signaling with heart failure risk estimated in MR analysis.

**Biomarker**	**IVs (SNPs)**	**OR (95% CI)**	***P*** **-value**
**IL-1β**	1	1.04 (0.86–1.25)	0.70
**IL-1ra**	2	0.95 (0.83–1.10)	0.51
Exclude rs4251961	1	1.00 (0.82–1.24)	0.95
**IL-6**	2	0.86 (0.81–0.91)	<0.001
Exclude rs643434	1	0.89 (0.77–1.03)	0.12
**sIL-6r**	1	0.96 (0.91–1.01)	0.14

*IL-1β, interleukin-1β; IL-1ra, interleukin-1 receptor antagonist; IL-6, interleukin-6; sIL-6r, soluble IL-6 receptor*.

## Discussion

In our MR analyses of a European population, our findings did not support an important etiological role of CRP and fibrinogen in HF development. Various sensitivity analyses supported our initial findings. In our study investigating IL-1 and IL-6 signaling, there was some evidence of an association between genetically determined IL-6 and HF risk but not for IL-1β, IL-1ra, or sIL-6r. However, this association became non-significant after excluding SNPs with pleiotropy.

Inflammation has been considered to contribute to the pathogenesis and progression of HF through various mechanistic pathways, such as cardiomyocyte apoptosis, cardiac fibrosis, and endothelial dysfunction (14). Activation of systemic inflammation has been widely reported in patients with HF as elevated levels of various markers, such as CRP and fibrinogen (37, 38). Evidence from observational cohort studies has shown that increased CRP and fibrinogen are independent risk factors for HF, suggesting that these inflammation biomarkers may play etiological roles in HF. However, given that the hemodynamic stress of HF itself can induce a state of sterile inflammation (39), whether the elevation of specific biomarkers of inflammation is a reflection of their involvement in disease pathogenesis or an epiphenomenon of HF remains unsolved. Meanwhile, the inherent limitations of conventional observational studies, such as residual confounding and reverse causation, limit the ability to ascertain causal inferences. Random control trials (RCTs) are the most powerful method to demonstrate the etiology hypothesis observed in epidemiological studies (40). However, it is difficult to implement RCTs because of rigorous research designs and expensive costs. In recent years, MR research has been acknowledged as the best alternative to RCTs given that it is a very reliable method that uses genetic variants inherited randomly from parents to infer a causal relationship between risk factors and diseases (40, 41). Previous MR studies have indicated that CRP was unlikely to be a causal factor for ischemic cardiovascular disease (42), and there was only very weak evidence of a causal effect of fibrinogen on coronary heart disease (CHD) (43). In our MR study, we analyzed the correlation between inflammatory biomarkers and HF risk with the aid of large-scale GWAS datasets. Our findings revealed that genetically elevated CRP and fibrinogen were not significantly associated with HF, suggesting that these inflammatory biomarkers may function as bystanders instead of causative factors in HF.

The “upstream” proinflammatory biomarkers IL-1 and IL-6 act as important initiators of inflammation and can trigger a cascade of inflammatory mediators, such as CRP and fibrinogen (44). Although evidence from preclinical and clinical studies suggested that the IL-1-IL-6 signaling pathway led to impaired systolic and diastolic cardiac function (45), clinical trials choosing these pathways as anti-inflammatory therapeutic targets have reported controversial results. Administration of anakinra, an IL-1 receptor antagonist, yielded beneficial effects on aerobic capacity improvement but did not reduce the length of hospital stay or rehospitalization in HF (14, 46). An RCT found a significant reduction in IL-6 and CRP concentrations in 267 patients treated with colchicine compared with those treated with placebo (47). However, there was no improvement in cardiac function or measures of LV remodeling in the colchicine group (47). Recent results from the Canakinumab Anti-Inflammatory Thrombosis Outcomes Study (CANTOS) provided evidence that targeting IL-1β with canakinumab significantly reduced major cardiovascular event rates, including HF hospitalizations (48), and that the magnitude of risk reduction was correlated with the magnitude of IL-6 reduction (44). Thus, canakinumab exhibited minimal improvement in patients who did not achieve substantial reductions in IL-6. Our MR analyses initially found a significant causal effect for IL-6 on HF but not for IL-1β, IL-1ra, or sIL-6r. The inverse association between genetically predicted levels of circulating IL-6 and HF was consistent with previous MR studies that focused on CHD (49, 50), indicating that IL-6 inhibition may be associated with lower risk of HF. However, after excluding an SNP with pleiotropy (rs643434), the association became non-significant. Nevertheless, the MR analysis likely reflects lifelong exposure to risk factors, which may explain the different effects between endogenous inhibition of IL-6 and exogenous blockade of IL-6 signaling. Therefore, it is possible that the administration of an IL-6 inhibitor under a specific condition may reduce HF risk.

A major strength of this study was the design of MR analysis based on the largest GWAS meta-analysis on HF, which can prevent reverse causation and the influence of potential confounders. We also conducted additional analyses excluding SNPs with potential pleiotropy, which can minimize the bias in causal effect estimates. In addition, we performed a comprehensive evaluation of causal inferences of inflammatory biomarkers in the IL-1-IL-6-CRP pathway, which may provide a better understanding of the role of this pathway in the pathogenesis of HF.

Our study had several limitations. First, we restricted the study population to European ancestry to reduce bias from population stratification. This restriction reduced the transferability to individuals with other genetic backgrounds. Second, because of the unavailability of individual data, we could not conduct analyses stratified by subtypes and severity of HF. Since observational studies suggested that a stronger association of inflammatory markers may exist in the context of HF with preserved EF (HFpEF), the etiological role of inflammation in specific subphenotypes requires future research. Third, we only investigated the causal relationship between certain inflammatory biomarkers and HF from a genetic point of view. Therefore, our results should be treated with caution, since the causal effect of SNP exposure on SNP outcome can be modified by compensatory processes during development, and the etiological roles of other inflammatory factors in HF need further exploration.

## Conclusion

Our MR analysis did not identify convincing evidence to support the causal relationship between inflammatory biomarkers, such as CRP and fibrinogen, or their upstream IL-1-IL-6 pathway with HF. Additional human and animal studies are needed to confirm our MR results further.

## Data Availability Statement

The original contributions presented in the study are included in the article/Supplementary Material, further inquiries can be directed to the corresponding author/s.

## Author Contributions

XL and SP designed this study and conducted the main analysis. BG, SC, GZ, YW, CG, JX, XL, XZ, and SL reviewed and edited the article. All authors contributed to the article and approved the submitted version.

## Funding

This work was supported by the National Natural Science Foundation of China (Grant No. 81970273).

## Conflict of Interest

The authors declare that the research was conducted in the absence of any commercial or financial relationships that could be construed as a potential conflict of interest.

## Publisher's Note

All claims expressed in this article are solely those of the authors and do not necessarily represent those of their affiliated organizations, or those of the publisher, the editors and the reviewers. Any product that may be evaluated in this article, or claim that may be made by its manufacturer, is not guaranteed or endorsed by the publisher.
