# Supplementary material for: Genetically Determined Inflammatory Biomarkers and the Risk of Heart Failure: A Mendelian Randomization Study
Source: Front Cardiovasc Med. 2021 Nov 22;8:734400. doi: 10.3389/fcvm.2021.734400 (PMC8645870; doi:10.3389/fcvm.2021.734400)
Supplement: Supplementary file 1 [file Data_Sheet_1.docx]

**Supplementary Material**

| **List of Supplementary Materials** | **Page** |
| --- | --- |
| Supplementary Table 1. Definitions of heart failure from 26 cohorts in Heart Failure Molecular Epidemiology for Therapeutic Targets (HERMES) Consortium. | 2-7 |
| Supplementary Table 2. Summary statistics for the single-nucleotide polymorphisms associated with exposure and their association with heart failure in the present Mendelian randomization study. | 8-9 |
| Supplementary Table 3. Mendelian randomization estimates of CRP and fibrinogen with heart failure after excluding SNPs with potential pleiotropy | 10 |
| Figure S1. Predefined decision tree for the selection of methods to evaluate associations using two-sample mendelian randomization analysis. |  |
| Figure S2. Mendelian randomization leave-one-out sensitivity analyses for C-reactive protein (CRP) and fibrinogen with heart failure. |  |
| Figure S3. After excluding pleiotropy single nucleotide polymorphisms (SNPs), the forest plot (A) and scatter plot (B) of CRP-associated SNPs potential effects on heart failure. IVW, inverse variance weighted. |  |
| Figure S4. After excluding pleiotropy SNPs, the forest plot (A) and scatter plot (B) of fibrinogen-associated SNPs potential effects on heart failure. |  |

**Supplementary Table 1.** Definitions of heart failure from 26 cohorts in Heart Failure Molecular Epidemiology for Therapeutic Targets (HERMES) Consortium.

| **Study Name** | **Study Design** | **HF cases/controls** | **Heart Failure Definition** |
| --- | --- | --- | --- |
| ARIC | Population-based prospective cohort study | 1,835/7,476 | Incident HF was defined as the first HF hospitalization or presence of HF code on death certificate since baseline visit through 2013. Discharge records and death certificates that showed a HF code in any position with International Classification of Diseases Code, Ninth Revision (ICD-9) code 428.x, and deaths with ICD-9/10 codes of either 428.x or I50 were considered as HF. Prevalent Heart Failure was ascertained at first visit. If the participant reported to have taken any medication for heart failure, or qualifies for the Gothenburg Criteria then the participant had prevalent heart failure at baseline. |
| BIOSTAT-CHF | Heart failure prospective cohort study | 1,561/954 | Heart failure was defined based on physician diagnosis, previous documented admission with heart failure requiring diuretic treatment, treatment with furosemide ≥20 mg/day or equivalent. |
| CHS | Population-based prospective cohort study | 1,123/2,145 | Incident HF events were identified by self-report or administrative data validated by physician’s review of medical records, as described in previous reports (PMID: 1669507). In brief, heart failure was defined on the basis of cardiomegaly and pulmonary edema on chest X-ray; or dilated ventricle and wail-motion abnormalities by echocardiography or contrast ventriculography; or congestive failure diagnosed by physician, plus receiving medical treatment (diuretic plus either digitalis, vasodilator or angiotensin converting enzyme inhibitor). |
| COGEN | Healthcare-based prospective cohort study | 636/2,215 | Inclusion criteria: patients ≥18 years, LVEF <40% or symptoms of clinical HF assessed by a physician including NYHA>1 Exclusion criteria: patients with cardiac valvular pathology (e.g. aortic stenosis), HTx, PAH or other structural heart disease |
| deCODE | Population-based prospective cohort study | 13,200/367,730 | Heart failure case status was assigned based on ICD-9 or ICD-10 codes for discharge diagnoses (ICD-10: I50 and subcodes, ICD-9: 428 and subcodes). |
| EGCUT | Population-based prospective cohort study | 2,699/10,709 | Heart failure status was assigned based on ICD-10: I50 and subcodes. |
| EPHESUS | Heart failure randomised controlled trial | 1,094/886 | Patients were recruited 3 to 14 days after acute myocardial infarction according to the following critera: acute myocardial infarction as documented according to standard criteria; left ventricular dysfunction as documented by a left ventricular ejection fraction of 40 percent or lower on echocardiography, radionuclide angiography, or angiography of the left ventricle after the index acute myocardial infarction and before randomization; and heart failure as documented by the presence of pulmonary rales, chest radiography showing pulmonary venous congestion, or the presence of a third heart sound. In patients with diabetes who met the criteria for left ventricular dysfunction after acute myocardial infarction, symptoms of heart failure did not have to be demonstrated, since such patients have an increased risk of cardiovascular events similar to that of nondiabetic patients with symptoms of heart failure. |
| EPIC-Norfolk | Population-based prospective cohort study | 1,687/17,631 | Heart failure definition was based on hospital admission or death record listing Heart failure code - ICD-10: I50 and subcodes |
| FHS | Population-based prospective cohort study | 476/3,290 | Criteria for defining heart failure in the FHS have been described previously (PMID: 5122894, 16837677). In brief, heart failure was considered to be present if two major or one major plus two minor criteria were present in the absence of an alternative explanation for the symptoms and signs. Major criteria are defined as paroxysmal nocturnal dyspnea, orthopnea, jugular venous distention, hepatojugular reflux, pulmonary rales, radiographic evidence of cardiomegaly, acute pulmonary edema, third heart sound, central venous pressure >16 cm of water, and weight loss >4.5 kg during first 5 days of treatment for suspected heart failure. Minor criteria are defined as bilateral ankle edema, nocturnal cough, dyspnea on ordinary exertion, hepatomegaly, pleural effusion, and heart rate >120 beats per minute. |
| FINRISK | Population-based prospective cohort study | 1,967/20,298 | Individuals with a diagnosis corresponding to heart failure in the nationwide hospital discharge or cause of death registers (ICD-10: I50, I110, I130 and I132; ICD-9: 4029B, 404, 4148, 428; ICD-7: 42700, 42710, 428) or special drug reimbursement for heart failure medications (requires a medical certificate that meets predefined criteria for heart failure). |
| GoDARTS | Population-based prospective cohort study | 568/3,811 | HF cases were defined by the presence of at least one of the following criteria: 1. Echocardiographic evidence of left ventricular systolic impairment and diuretic prescription 2. Admission to hospital with HF and receipt of a loop diuretic prescription Patients who were never prescribed a loop diuretic were not classified as HF cases. Date of HF diagnosis was taken as either the date of the earliest echocardiogram or the date of the earliest admission to hospital for HF. |
| GRADE | Prospective cohort study | 1,060/7,709 | Inclusion criteria were: patients who were ≥18 years of age with a diagnosis of at least moderate systolic left ventricular dysfunction (EF ≤30%), and who had an ICD at the University of Pittsburgh Medical Center, Emory University Medical Center, Massachusetts General Hospital, Ohio State University Medical Center, Mid-Ohio Cardiology or the Pittsburgh Veterans Affairs Medical Center. Subjects were excluded if they had intractable Class IV heart failure, and conditions (other than HF) that were expected to limit survival to less than 6 months. |
| LURIC | Population-based prospective cohort study | 1,007/2,054 | A clinical diagnosis of (left) heart failure was defined by the combined presence of symptoms of dyspnea on exertion and cardiac disease with impaired left ventricular function. Left ventricular dysfunction or impaired left ventricular function was defined by imaging techniques, such as echocardiography and left ventricular angiography, and graded semi-quantitatively into normal, minimal, moderate and severe impairment of left ventricular pump function. Based on the underlying cardiac disease, heart failure/left ventricular dysfunction was either of ischaemic (in case of CAD) or of non-ischaemic origin (dilated cardiomyopathy being the most frequent non-CAD disease). Mis- or underreporting of heart failure and/or left ventricular dysfunction was avoided in LURIC since virtually all LURIC participants, except for family members without coronary angiography, underwent echocardiography and left ventricular angiography. |
| MDCS | Population-based prospective cohort study | 755/7,590 | Heart failure was ascertained from the Swedish Hospital Discharge Register using diagnosis codes 427.00, 427.10, and 428.99 for International Classification of Diseases-8th Revision (ICD-8), 428 for the 9th Revision (ICD-9), and I50 and I11.0 for the 10th Revision (ICD-10) as primary diagnosis, according to a previous validation study (PMID:15916919). |
| PHFS | Heart failure prospective cohort study | 2,206/4,523 | Prevalent heart failure diagnosed by a heart failure cardiologist based on clinical evaluation and cardiac imaging |
| PIVUS | Population-based prospective cohort study | 116/833 | The medical records for all individuals with heart failure diagnosis in any position in the Swedish hospital discharge register were reviewed by two physicians who were blinded to the baseline data. They classified the cases as definite, questionable, or miscoded according to the European Society of Cardiology recommendations. They considered ICD heart failure codes 427.00, 427.10, 428 (ICD-9), I50 (ICD-10) and hypertensive heart disease with heart failure, I11.0 (ICD-10) as possible diagnosis of heart failure. For further details see PMID:15916919. |
| PREVEND | Population-based prospective cohort study | 163/3,486 | Heart failure cases were ascertained using criteria in accordance with the Heart Failure Guidelines of the European Society of Cardiology (ESC). In- and outpatient files were inspected for the presence of heart failure at baseline and for new onset heart failure, by recording signs, symptoms, and objective evidence of heart failure. In total, 586 individual cases were identified as suspected heart failure. An endpoint adjudication committee of seven independent experts evaluated all suspected cases of new onset heart failure. Each case was validated by two different experts by reviewing anonymized clinical charts, hospitalization, and physician office records in order to ascertain the incidence of heart failure. In case of consensus, patients were classified as ‘definite new onset heart failure’, ‘definite no new onset heart failure’, or ‘definite heart failure, with date of onset before time of recruitment. In case of difference of opinion about an individual case, the committee made a joint decision. |
| PROSPER | Randomised-controlled trial | 211/5,033 | Cases were defined by hospitalization for heart failure with a definition based on a combination of symptoms (e.g. shortness of breath) and signs, including chest radiograph with fluid congestion or echocardiogram with severely diminished LV function. All outcomes were adjudicated by an expert committee blinded to randomized study medication and using pre‐defined criteria. |
| Regeneron/Geisinger | Population-based prospective cohort study | -/- | Heart failure status was assigned based on ICD-10: I50 and subcodes. |
| Rotterdam study 1 | Population-based prospective cohort study | 876/3,807 | Prevalent heart failure at baseline was assessed using a validated score based on the European Society of Cardiology recommendation, identified from hospital discharge diagnoses, and restropective medical records screening. Cases of incident heart failure were obtained by continuously monitoring participants for the occurrence of heart failure during follow-up through general practitioners records and hospital discharge diagnoses. The date of incident heart failure was defined as the day of the first occurrence of symptoms suggestive of heart failure, obtained from the medical records, or the day of receipt of a first prescription for a loop diuretic or an ACEinhibitor indicated for treatment of heart failure, whichever came first. The diagnosis of heart failure was classified as definite, probable, possible, or unlikely in accordance with the criteria from the European Society of Cardiology. Potential cases were ascertained by two research physicians and verified by a cardiologist. Only definite and probable cases were considered in the analyses. |
| SHIP | Population-based prospective cohort study | 352/1,126 | For these analyses heart failure was defnied according to a modified Rotterdam definition (PMID: 10213348). Prevalent HF cases in SHIP were defined as having history of HF (either chest pain during exercise, bypass, heart transplant, atrial flutter or fibrillation, LV hypertophy in individuals aged 45 or older, known MI) and HF symptoms (dyspnoe at exercise or swollen legs at evening) that were not related to bronchitis (bronchitis that occured recently or during the last 12 months). |
| SOLID | Randomised-controlled trial | 186/9,172 | Heart failure status at enrolment was identified from medical record with no specific definition. I HF hospitalizations adjudicated during follow up were defined as admission to hospital or attendance at an acute health care facility for administration of intravenous diuretic treatment, escalation of diuretic doses, and/or inotropes. Confirmation of heart failure diagnosis was obtained by chest imaging demonstrating pulmonary congestion or edema, or, in patients without available chest imaging, at least one of the following: Pulmonary edema, (i.e. rales >1/3 up the lung fields thought to be of cardiac causes), pulmonary capillary wedge pressure >18 mmHg or BNP >500 pg/ml (or NT-terminal prohormone BNP >2500 pg/ml). |
| TwinGene | Population-based prospective cohort study | 604/5,176 | Heart failure status was assigned based on ICD-10: I50; ICD-8 and ICD-9 428 |
| UK Biobank | Population-based prospective cohort study | 6,504/387,652 | Individuals with self-reported "HF/pulmonary edema" or "cardiomyopathy"; or who carry an International Classification of Diseases (ICD)-10 or ICD-9 billing code for heart/ventricular failure or cardiomyopathy (ICD-10: I11.0, I13.0, I13.2, I25.5, I42.0, I42.5, I42.8, I42.9, I50.0, I50.1, I50.9; ICD-9: 4254, 4280, 4281, 4289). Individuals with self-reported or an ICD-10 based classification of hypertrophic cardiomyopathy were excluded. |
| ULSAM | Population-based prospective cohort study | 288/891 | The medical records for all individuals with heart failure diagnosis in any position in the Swedish hospital discharge register were reviewed by two physicians who were blinded to the baseline data. They classified the cases as definite, questionable, or miscoded according to the European Society of Cardiology recommendations. They considered ICD heart failure codes 427.00, 427.10, 428 (ICD-9), I50 (ICD-10) and hypertensive heart disease with heart failure, I11.0 (ICD-10) as possible diagnosis of heart failure. For details, please see Ingelsson E, et al Eur J Heart Fail. 2005 Aug;7(5):787-91 |
| WGHS | Population-based prospective cohort study | 479/22,791 | Heart failure cases were ascertained by cardiologists from medical records. Cases of incident nonfatal HF were confirmed if either the Framingham Heart Study (mainly physical examination and radiographic data) or Cardiovascular Health Study criteria (predominantly based on the treating physician's diagnosis and use of specific therapy) were met. Fatal HF cases included those not identified as a case of HF prior to death and classified into “Definite” or “Probable” fatal HF based on medical records and death certificate with next-of-kin or physician confirmation. |

**Supplementary Table 2.** Summary statistics for the single-nucleotide polymorphisms associated with exposure and their association with heart failure in the present Mendelian randomization study.

| **Exposure** | **SNP** | **Chr** | **Nearby gene** | **EA** | **EAF** | **Exposure** | | | **Outcome** | | |
| --- | --- | --- | --- | --- | --- | --- | --- | --- | --- | --- | --- |
|  |  |  |  |  |  | **Beta** | **SE** | ***P* value** | **Beta** | **SE** | ***P* value** |
| **CRP** | **rs10512597** | **17** | **CD300LF** | **T** | **0.18** | **-0.037** | **0.005** | **4.44×10^-14^** | **-0.0037** | **0.0117** | **0.7532** |
|  | **rs1051338** | **10** | **LIPA** | **G** | **0.31** | **0.024** | **0.004** | **2.27×10^-09^** | **0.0303** | **0.0085** | **0.000366** |
|  | **rs10521222** | **16** | **SALL1** | **C** | **0.05** | **0.104** | **0.011** | **2.06×10^-22^** | **-0.0192** | **0.0188** | **0.3073** |
|  | **rs10832027** | **11** | **ARNTL** | **G** | **0.33** | **-0.026** | **0.004** | **4.43×10^-12^** | **-0.013** | **0.0085** | **0.1263** |
|  | **rs10838687** | **11** | **MADD** | **G** | **0.22** | **-0.031** | **0.004** | **9.12×10^-13^** | **-0.0032** | **0.0096** | **0.7412** |
|  | **rs10925027** | **1** | **NLRP3** | **T** | **0.4** | **0.036** | **0.004** | **4.25×10^-21^** | **0.0058** | **0.008** | **0.4678** |
|  | **rs12202641** | **6** | **FRK** | **T** | **0.39** | **-0.023** | **0.004** | **3.00×10^-10^** | **-0.0056** | **0.008** | **0.4849** |
|  | **rs1260326** | **2** | **GCKR** | **T** | **0.39** | **0.073** | **0.004** | **2.72×10^-92^** | **0.0087** | **0.0081** | **0.2797** |
|  | **rs12960928** | **18** | **MC4R** | **C** | **0.27** | **0.024** | **0.004** | **1.91×10^-09^** | **0.0198** | **0.0088** | **0.02385** |
|  | **rs12995480** | **2** | **TMEM18** | **T** | **0.17** | **-0.031** | **0.005** | **1.24×10^-10^** | **-0.0313** | **0.0105** | **0.002725** |
|  | **rs13233571** | **7** | **BCL7B** | **C** | **0.12** | **0.057** | **0.005** | **2.95×10^-25^** | **0.0092** | **0.0122** | **0.4523** |
|  | **rs13409371** | **2** | **IL1F10** | **A** | **0.43** | **0.048** | **0.004** | **5.07×10^-36^** | **-0.0072** | **0.0093** | **0.4398** |
|  | **rs1441169** | **2** | **IKZF2** | **G** | **0.53** | **-0.025** | **0.004** | **2.27×10^-11^** | **0.0011** | **0.0078** | **0.8911** |
|  | **rs1490384** | **6** | **C6orf173** | **T** | **0.51** | **-0.025** | **0.004** | **2.65×10^-12^** | **-0.0034** | **0.0079** | **0.6661** |
|  | **rs1582763** | **11** | **MS4A4A** | **A** | **0.37** | **-0.022** | **0.004** | **2.37×10^-09^** | **0.0171** | **0.0081** | **0.03606** |
|  | **rs17658229** | **5** | **DUSP1** | **C** | **0.05** | **0.056** | **0.01** | **5.50×10^-09^** | **-0.0257** | **0.0188** | **0.1701** |
|  | **rs178810** | **17** | **NCOR1** | **T** | **0.56** | **0.02** | **0.004** | **2.95×10^-08^** | **0.0204** | **0.0079** | **0.009406** |
|  | **rs1800961** | **20** | **HNF4A** | **C** | **0.03** | **0.112** | **0.011** | **4.63×10^-23^** | **-0.0097** | **0.0215** | **0.6509** |
|  | **rs1805096** | **1** | **LEPR** | **G** | **0.39** | **0.104** | **0.004** | **2.17×10^-183^** | **0.002** | **0.0081** | **0.8056** |
|  | **rs1880241** | **7** | **IL6** | **G** | **0.48** | **-0.028** | **0.004** | **8.41×10^-14^** | **-0.0134** | **0.0078** | **0.08546** |
|  | **rs2064009** | **8** | **TRPS1** | **C** | **0.42** | **-0.027** | **0.004** | **2.28×10^-14^** | **-0.0192** | **0.008** | **0.01624** |
|  | **rs2239222** | **14** | **RGS6** | **G** | **0.36** | **0.035** | **0.004** | **9.87×10^-20^** | **-0.004** | **0.0083** | **0.6269** |
|  | **rs2315008** | **20** | **ZGPAT** | **T** | **0.31** | **-0.023** | **0.004** | **5.36×10^-10^** | **0.0022** | **0.0084** | **0.7931** |
|  | **rs2352975** | **3** | **TRAIP** | **C** | **0.3** | **0.025** | **0.004** | **6.43×10^-10^** | **0.0187** | **0.0086** | **0.02905** |
|  | **rs2710804** | **7** | **KIAA1706** | **C** | **0.37** | **0.021** | **0.004** | **1.30×10^-08^** | **-0.014** | **0.0082** | **0.08882** |
|  | **rs2794520** | **1** | **CRP** | **C** | **0.33** | **0.182** | **0.004** | **4.17×10^-523^** | **1.00×10^-04^** | **0.0083** | **0.988** |
|  | **rs2836878** | **21** | **DSCR2** | **G** | **0.27** | **0.043** | **0.004** | **7.71×10^-26^** | **-0.0024** | **0.0089** | **0.7889** |
|  | **rs2852151** | **18** | **PTPN2** | **A** | **0.4** | **0.025** | **0.004** | **1.36×10^-11^** | **-0.0078** | **0.0079** | **0.3235** |
|  | **rs2891677** | **8** | **NSMCE2** | **C** | **0.46** | **-0.02** | **0.004** | **1.59×10^-08^** | **0.0029** | **0.0078** | **0.7098** |
|  | **rs340005** | **15** | **RORA** | **A** | **0.38** | **0.03** | **0.004** | **1.01×10^-15^** | **2.00×10^-04^** | **0.0081** | **0.9782** |
|  | **rs4092465** | **18** | **ONECUT2** | **A** | **0.35** | **-0.027** | **0.004** | **3.11×10^-10^** | **-4.00×10^-04^** | **0.0084** | **0.9582** |
|  | **rs4129267** | **1** | **IL6R** | **C** | **0.39** | **0.088** | **0.004** | **1.20×10^-129^** | **0.0123** | **0.008** | **0.124** |
|  | **rs4246598** | **2** | **FABP1** | **A** | **0.46** | **0.022** | **0.004** | **5.11×10^-10^** | **-0.0022** | **0.0079** | **0.7853** |
|  | **rs4420638** | **19** | **APOC1** | **A** | **0.18** | **0.229** | **0.006** | **1.23×10^-305^** | **0.005** | **0.0106** | **0.6377** |
|  | **rs469772** | **1** | **ZNF644** | **T** | **0.19** | **-0.031** | **0.005** | **5.54×10^-12^** | **-0.0081** | **0.0099** | **0.4126** |
|  | **rs4841132** | **8** | **PPP1R3B** | **G** | **0.09** | **0.065** | **0.006** | **2.00×10^-25^** | **0.006** | **0.0143** | **0.6733** |
|  | **rs6001193** | **22** | **TOMM22** | **G** | **0.35** | **-0.028** | **0.004** | **6.53×10^-14^** | **0.0057** | **0.0082** | **0.4852** |
|  | **rs643434** | **9** | **ABO** | **A** | **0.37** | **0.023** | **0.004** | **1.02×10^-09^** | **0.0406** | **0.0082** | **8.03×10^-07^** |
|  | **rs7310409** | **12** | **HNF1A** | **G** | **0.39** | **0.137** | **0.004** | **2.54×10^-299^** | **-0.0319** | **0.0081** | **7.90×10^-05^** |
|  | **rs9271608** | **6** | **HLA-DQA1** | **G** | **0.22** | **0.042** | **0.005** | **2.33×10^-17^** | **-0.0032** | **0.0111** | **0.7758** |
|  | **rs9284725** | **2** | **IL1R1** | **C** | **0.24** | **0.027** | **0.004** | **7.34×10^-11^** | **-0.0026** | **0.0093** | **0.7806** |
| **Fibrinogen** |  |  |  |  |  |  |  |  |  |  |  |
|  | **rs10157379** | **1** | **NLRP3** | **T** | **0.62** | **0.01** | **0.001** | **1.15×10^-19^** | **0.0053** | **0.0081** | **0.5121** |
|  | **rs10226084** | **7** | **SNx13** | **T** | **0.52** | **-0.007** | **0.001** | **5.05×10^-10^** | **0.0078** | **0.0078** | **0.3228** |
|  | **rs10512597** | **17** | **CD300LF** | **T** | **0.18** | **-0.008** | **0.001** | **9.92×10^-09^** | **-0.0037** | **0.0117** | **0.7532** |
|  | **rs12712127** | **2** | **IL1R1** | **A** | **0.41** | **0.006** | **0.001** | **2.72×10^-08^** | **4.00×10^-04^** | **0.0081** | **0.9595** |
|  | **rs1476698** | **2** | **FARP2** | **A** | **0.65** | **0.007** | **0.001** | **2.24×10^-09^** | **-0.0112** | **0.0081** | **0.168** |
|  | **rs16844401** | **4** | **HGFAC** | **A** | **0.08** | **0.015** | **0.003** | **1.74×10^-08^** | **0.051** | **0.0164** | **0.001945** |
|  | **rs1800789** | **4** | **FGB** | **A** | **0.21** | **0.031** | **0.001** | **1.68×10^-127^** | **-0.0034** | **0.0099** | **0.7344** |
|  | **rs1938492** | **1** | **LEPR** | **A** | **0.62** | **0.008** | **0.001** | **5.28×10^-14^** | **0.0016** | **0.008** | **0.8414** |
|  | **rs2106854** | **5** | **C5orf56** | **T** | **0.21** | **-0.019** | **0.001** | **1.72×10^-48^** | **0.0109** | **0.0097** | **0.2604** |
|  | **rs2286503** | **7** | **T0MM7** | **T** | **0.36** | **-0.006** | **0.001** | **6.88×10^-09^** | **-0.0166** | **0.0082** | **0.04225** |
|  | **rs4129267** | **1** | **IL6R** | **T** | **0.39** | **-0.011** | **0.001** | **5.97×10^-27^** | **-0.0123** | **0.008** | **0.124** |
|  | **rs434943** | **14** | **ACTN1** | **A** | **0.31** | **0.007** | **0.001** | **1.08×10^-08^** | **0.0124** | **0.0085** | **0.1462** |
|  | **rs4817986** | **21** | **PSMG1** | **T** | **0.28** | **-0.008** | **0.001** | **2.46×10^-11^** | **2.00×10^-04^** | **0.0087** | **0.9835** |
|  | **rs6010044** | **22** | **SHANK3** | **A** | **0.8** | **-0.008** | **0.001** | **3.41×10^-08^** | **-9.00×10^-04^** | **0.0096** | **0.9261** |
|  | **rs6734238** | **2** | **IL1F10** | **A** | **0.58** | **-0.009** | **0.001** | **5.77×10^-19^** | **-0.0034** | **0.008** | **0.6733** |
|  | **rs715** | **2** | **CPS1** | **T** | **0.68** | **0.009** | **0.001** | **1.98×10^-11^** | **0.0137** | **0.0087** | **0.1125** |
|  | **rs7204230** | **16** | **CHD9** | **T** | **0.7** | **0.008** | **0.001** | **1.18×10^-10^** | **0.0064** | **0.0084** | **0.4443** |
|  | **rs7896783** | **10** | **JMJD1C** | **A** | **0.48** | **-0.01** | **0.001** | **8.90×10^-22^** | **0.017** | **0.0079** | **0.03169** |
|  | **rs7968440** | **12** | **DIP2B** | **A** | **0.64** | **0.006** | **0.001** | **2.74×10^-08^** | **0.0237** | **0.0082** | **0.003865** |
| **IL-1b** |  |  |  |  |  |  |  |  |  |  |  |
|  | **rs6917603** | **6** | **HLA locus** | **C** | **0.25** | **-0.163** | **0.023** | **1.76×10^-12^** | **-0.006** | **0.0154** | **0.6978** |
| **IL-1ra** |  |  |  |  |  |  |  |  |  |  |  |
|  | **rs4251961** | **2** | **ILRN** | **C** | **0.32** | **-0.082** | **0.009** | **2.80****×10^-21^** | **0.0078** | **0.0082** | **0.3416** |
|  | **rs6759676** | **2** | **IL1F10** | **C** | **0.44** | **0.075** | **0.009** | **1.73×10^-17^** | **5.00×10^-04^** | **0.008** | **0.9478** |
| **IL-6** |  |  |  |  |  |  |  |  |  |  |  |
|  | **rs643434** | **9** | **ABO** | **A** | **0.26** | **-0.258** | **0.026** | **2.69×10^-21^** | **0.0406** | **0.0082** | **8.03×10^-07^** |
|  | **rs4129267** | **1** | **IL6R** | **T** | **0.26** | **0.109** | **0.02** | **2.36×10^-08^** | **-0.0123** | **0.008** | **0.124** |
| **sIL-6r** |  |  |  |  |  |  |  |  |  |  |  |
|  | **rs2228145** | **1** | **IL6R** | **C** | **0.36** | **0.295** | **0.015** | **2.44×10^-88^** | **-0.0117** | **0.0081** | **0.1489** |

CRP, C-reactive protein; IL-1b, Interleukin-1b; IL-1ra, Interleukin-1 receptor antagonist; sIL-6r: soluble interleukin-6 receptor; SNP, single-nucleotide polymorphism; Chr, chromosome; EA, effect allele; EAF, effect allele frequency; SE, standard error

**Supplementary Table 3.** Mendelian randomization (MR) estimates of CRP and fibrinogen with heart failure after excluding SNPs with potential pleiotropy

| Phenotype and methods | IVs (SNPs) | OR (95% CI) | *P*-value | Q-statistics | *P*h |
| --- | --- | --- | --- | --- | --- |
| **CRP** | | | | | |
| IVW | 13 | 1.02 (0.93-1.12) | 0.65 | 24.17 | 0.019 |
| **Weighted median** | 13 | 1.01 (0.93-1.09) | 0.83 |  |  |
| MR-Egger | 13 | 1.03 (0.89-1.20) | 0.67 |  |  |
| MR-PRESSO | 13 | 1.02(0.93-1.12) | 0.65 |  |  |
| **Fibrinogen** | | | | | |
| **IVW** | 11 | 1.18 (0.77-1.81) | 0.46 | 10.54 | 0.39 |
| Weighted median | 11 | 0.96 (0.56-1.65) | 0.89 |  |  |
| MR-Egger | 11 | 0.54 (0.25-1.20) | 0.17 |  |  |
| MR-PRESSO | 11 | 1.18(0.77-1.81) | 0.46 |  |  |

IV, instrumental variable; OR, odds ratio; CI, confidence interval; *P*h: P value for heterogeneity

**Supplementary Figure Legends**

**Figure S1.** Predefined decision tree for the selection of methods to evaluate associations using two-sample mendelian randomization.

**Figure S2.** Mendelian randomization leave-one-out sensitivity analyses for C-reactive protein (CRP) and fibrinogen with heart failure.

**Figure S3.** After excluding pleiotropy single nucleotide polymorphisms (SNPs), the forest plot (A) and scatter plot (B) of CRP-associated SNPs potential effects on heart failure. IVW, inverse variance weighted.

**Figure S4.** After excluding pleiotropy SNPs, the forest plot (A) and scatter plot (B) of fibrinogen-associated SNPs potential effects on heart failure.
