# Supplementary figures and images for: Genetically Determined Inflammatory Biomarkers and the Risk of Heart Failure: A Mendelian Randomization Study
Source: Front Cardiovasc Med. 2021 Nov 22;8:734400. doi: 10.3389/fcvm.2021.734400 (PMC8645870; doi:10.3389/fcvm.2021.734400)

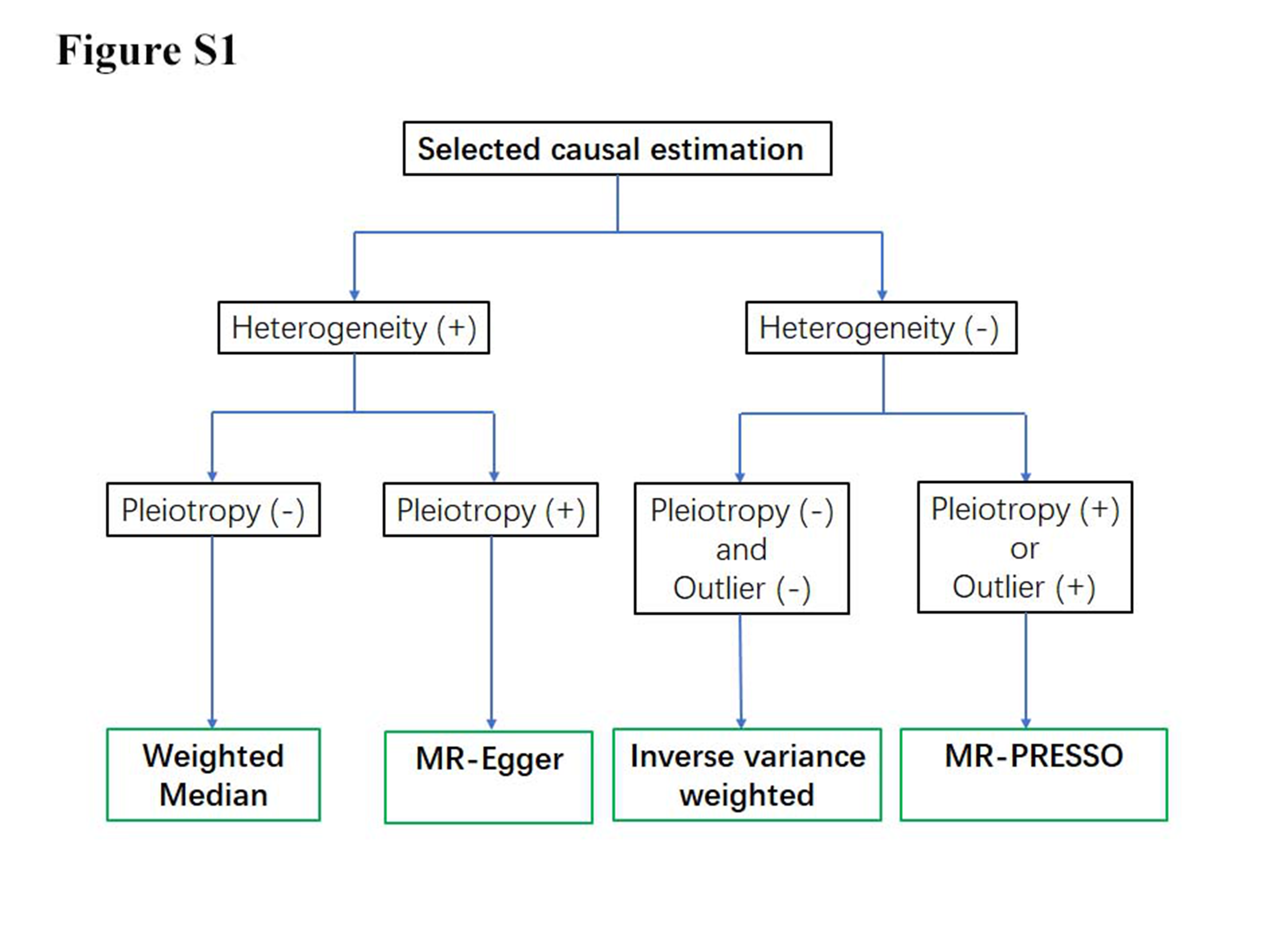

Supplement: Supplementary file 2 [file Image_1.TIF]

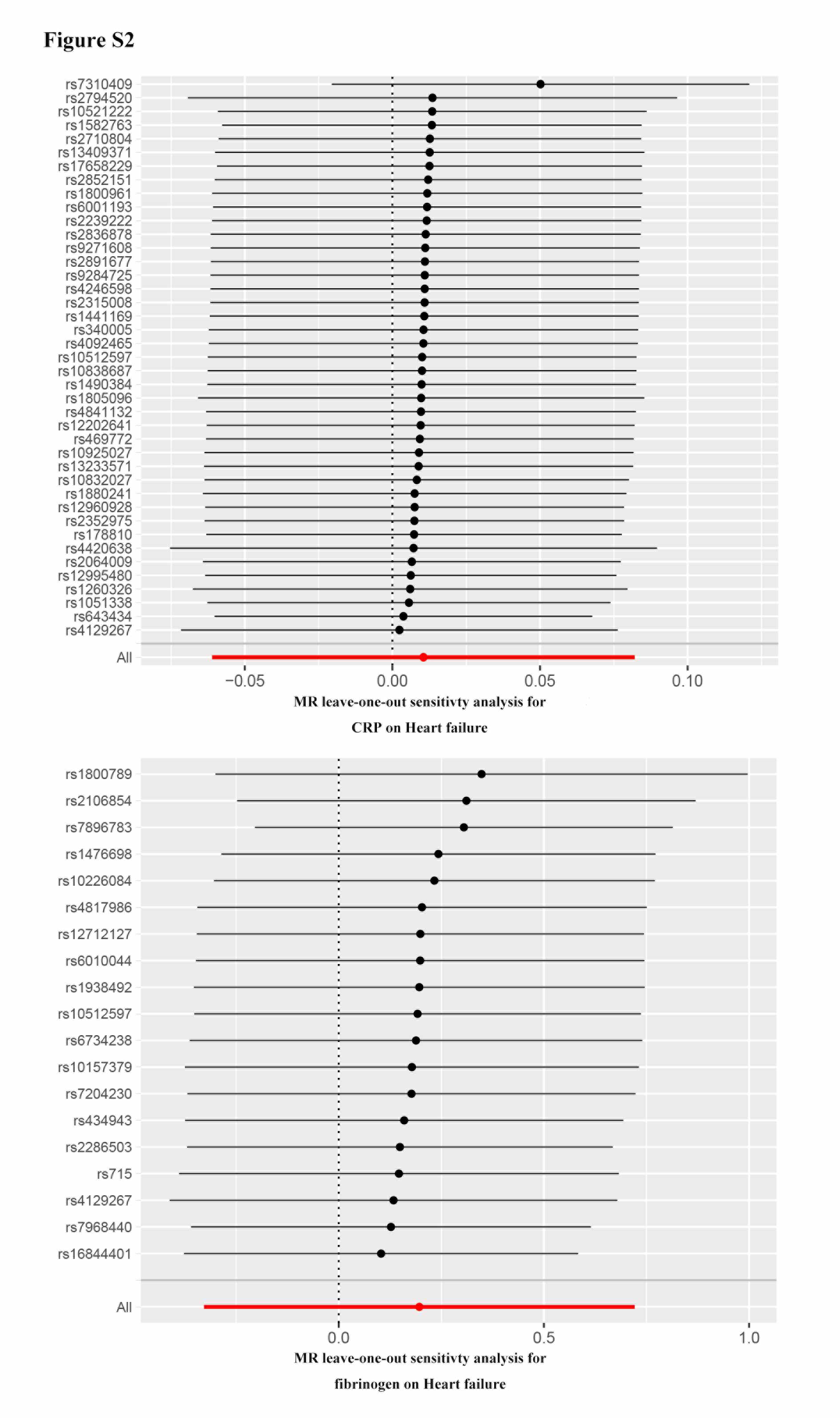

Supplement: Supplementary file 3 [file Image_2.TIF]

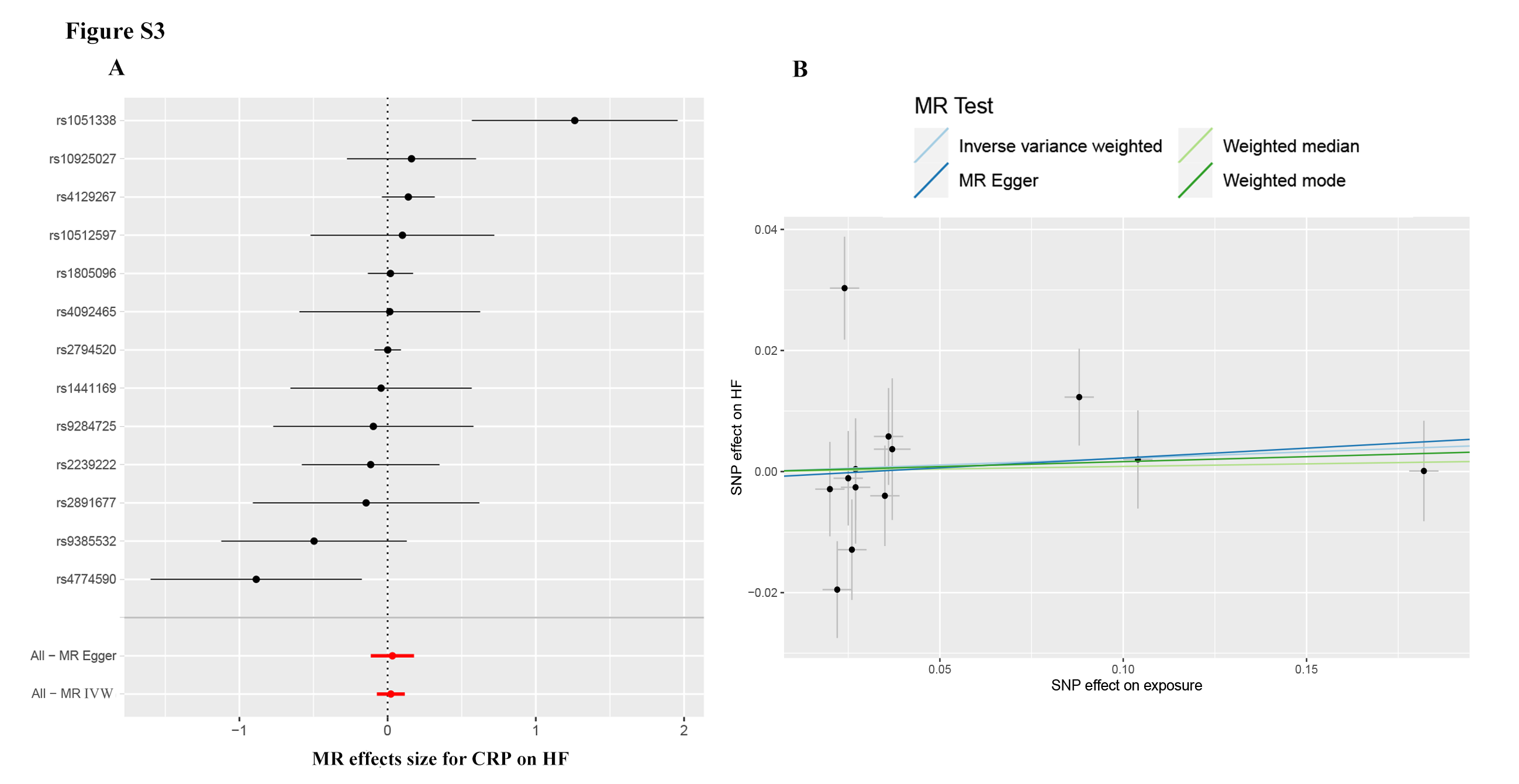

Supplement: Supplementary file 4 [file Image_3.TIF]

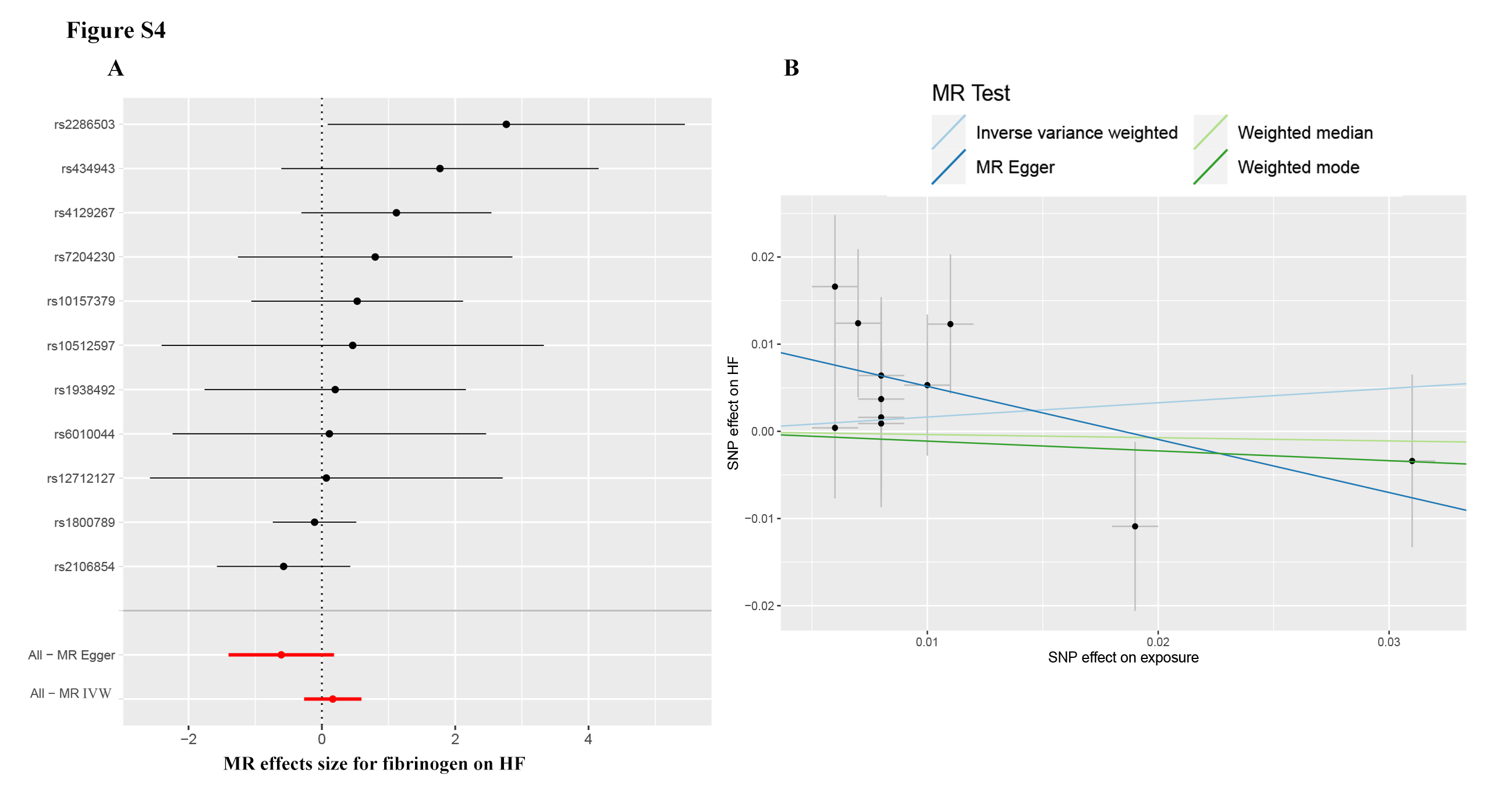

Supplement: Supplementary file 5 [file Image_4.TIF]
